# Modern Sociology

**Published:** 1899-02-18

**Authors:** 


### Modern Sociology.

### HABITUAL DRUNKARDS.

By Francis Scougall.

A letter just issued by the Home Secretary, and addressed to Judges, Chairmen of Quarter Sessions, and Recorders, in explanation of the new Act passed last Session for the reformatory treatment of habitual drunkards, deals with one of the most important measures—I say it emphatically—that has ever been undertaken by the Legislature for the amelioration of our people's condition generally. What the curse of drunkenness has long been to this country is patent to all. One has no need to speak of ruined lives, of desecrated homes, children neglected, wives ill-treated, or honest men driven to destitution and reckless despair by drunken wives—all the hideous forms of misery which are attributable to this cause are known to everyone, and might alone suffice to render us deeply thankful for the passing of the Bill which attempts to grapple with this gigantic evil; but there is a specially dark and hopeless stage in the Inferno of drink with which the Home Secretary's letter mainly deals. Those whose business it is to go down into the deep gulf of prison life are brought face to face with the fact that, in the great majority of cases, drink has been the primary cause of the crimes which have brought convicts within these ignominious walls. If the unlimited powers of getting drunk which our teeming public-houses offer to the populations of our large cities and country districts could be abolished, it is certain that three-fourths of our prisons might be closed altogether. The Home Secretary's letter deals, however, with the facts as they exist, and he explains the two different classes of individuals for whom the provisions of the Act have been formed: First, it is enacted that habitual drunkards, those that is who have been convicted four times within one year of certain offences specified in the Act, may be sent to an inebriate reformatory, and detained there for a period of three years. Public opinion may possibly consider so lengthened a detention for the cure of a fatal habit, which is not in itself definitely criminal, to be too severe a sentence; but it is, in fact, an exercise of the greatest charity towards the habitual drinker, as all experience goes to prove that a lesser period would in most cases be utterly insufficient to effect the good result desired for the inebriates themselves, as well as for all who come in contact with them. Sir M. W. Ridley proceeds to explain that it is not intended, in the first instance, that these reformatory institutions should be established by the central Government, but by local authorities, religious or philanthropic associations, or by the public-spirited efforts of wealthy individuals; although, in order to encourage such private agencies, substantial contributions would be made from the national funds—as much as 10s. 6d. for inmates in certain cases, or 16s. 6d. for others of a different stamp. On this subject, however, the Home Secretary adds, after dealing with the details of the present plan, that should experience prove it to be desirable to establish a State reformatory he would be prepared to ask for the necessary funds from the Government. The Act, as it stands at present, does not come into active operation till four weeks after the meeting of Parliament on February 7th, but he has already been in communication with the persons who are prepared to undertake the charge of such institutions as responsible managers, and he has received satisfactory assurances that by the time the Act is enforced reformatories of the kind required will be ready for the reception of inmates. Sir M. W. Ridley then proceeds to explain that the new Act is designed by Parliament to replace the system of short sentences of imprisonment or fines, which has hitherto been the only method possessed by courts of summary jurisdiction in dealing with the special offences of drunkenness set forth in the first schedule of the Act, and which has been found simply useless in the case of confirmed inebriates. He requests the judges and other authorities to note, that under this Act they have power to order the detention for as much as three years of all habitual drunkards as above defined; but he adds that a system of licensing or probationary discharge will be applied to each case according as the individual circumstances will allow. Detention in these institutions is carefully explained to be not of a *punitive* but of a reformatory character, and the treatment carried on during detention will be followed by a term of probationary freedom under licence, which cannot be successfully carried out, even in favourable cases, under a period varying from eighteen months to two years.

The Home Secretary proceeds to explain that the second class of offenders who come within the provisions of the Act are persons who have been convicted of crimes punishable by penal servitude or imprisonment. In cases where the Court is satisfied that drunkenness was a primary or contributory cause of the offence, and the jury find that the criminal is an habitual drunkard, such persons may be sentenced for a period not exceeding three years to a certified reformatory or State institution, and the committal may either be in addition to any other sentence, or in substitution for it. This department, he states, has been met by the grave initial difficulty, that it has not been possible to form an estimate of the number of persons for whom provision would have to be made in a State reformatory; it is already certain that drunkards, repeatedly convicted of the minor crimes set out in the schedule of the Act, and who are, therefore, qualified for committal to a certified reformatory, number many hundreds throughout the country. There might have been one ready means by which a sufficiently extensive State reformatory could be provided, by simply adapting one of the large existing prisons for the purpose, but the Home Secretary has most wisely put an absolute veto on any such idea. He found that the requirements of a reformatory, both as regards suitable arrangements for work and recreation and other provisions for the welfare of the inmates, would be so entirely opposed to the system necessary in a prison that it would be impossible to convert the one into the other. Certified reformatories will, however, be ready when the Act comes into operation, and I confidently expect the speedy establishment of a State institution for the purpose. We may properly hail the passing of the new Act as one of the most salutary and valuable efforts for the good of our country which has ever been made by the Imperial Government.

